# *COVID-19 Stats:* COVID-19* and Influenza^†^ Discharge Diagnoses as a Percentage of Emergency Department (ED) Visits,^§^ by Year — United States, June 2018–March 2021

**DOI:** 10.15585/mmwr.mm7015a7

**Published:** 2021-04-16

**Figure:**
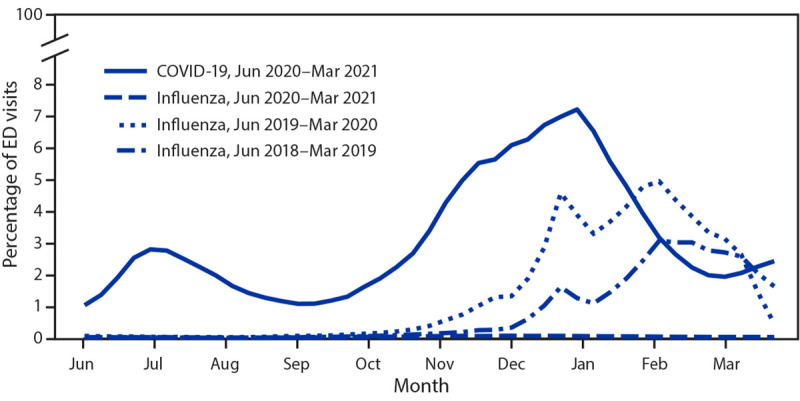
In late June 2020, the percentage of ED visits for COVID-19 increased and reached a peak of 2.8% of all ED visits in early July before declining through August. This decline was followed by a larger and more prolonged increase beginning in September 2020 that reached a peak (7.2%) in early January 2021. Influenza activity generally begins in October with increased activity throughout the winter months. By the beginning of February 2018, the percentage of ED visits for influenza reached 3.1%, and by the beginning of February 2019, reached 5.0%. During June 2020–March 2021, ED visits for influenza accounted for less than 0.1% of all visits.

**Source:** National Syndromic Surveillance Program, June 2018–March 2021. https://www.cdc.gov/nssp/index.html

